# The Value of Hybrid Teledermatology in German Prisons: Analysis of Routine Telemedical Data

**DOI:** 10.1089/tmj.2022.0467

**Published:** 2023-11-10

**Authors:** Brigitte Stephan, Gefion Girbig, Matthias Augustin, Eva Blozik, Martin Scherer, Natalia Kirsten, Marina Otten

**Affiliations:** ^1^ Institute for Health Services Research in Dermatology and Nursing (IVDP), University Medical Center Hamburg-Eppendorf (UKE), Hamburg, Germany.; ^2^ A+ Videoclinic GmbH, Munich, Germany.; ^3^ Institute of Primary Care, University Hospital of Zurich, Zurich, Switzerland.; ^4^ Department of General Practice and Primary Care, University Medical Center Hamburg-Eppendorf (UKE), Hamburg, Germany.

**Keywords:** telemedicine, prisoner, dermatology, needs, barriers

## Abstract

**Introduction:**

German prisons face organizational and time-consuming difficulties in access to medical specialties. Since 2019, our institute offers interdisciplinary video consultations with spatially independent dermatological support for German prisons.

**Methods:**

Documentation of* n* = 200 consultations between February 2020 and July 2021 with retrospective analysis of dermatological conditions and consultation requests.

**Results:**

Most cases (98.0%; 196 of 200) were performed during a regular weekly teleclinic and only few cases on urgent demand. The average duration of the skin disease before request for consultation was 10.3 ± 26.9 months (mean ± standard deviation), the majority had first onset of their disease or acute recurrence of previously known skin diseases. With respect to medical complaints, 39.7% of patients reported severe itch and 7.7% indicated severe pain. For most cases (84.0%), topical treatment and for almost one-third (32.5%) we recommended systemic treatment. The predominant number of cases was only presented once (92.0%) and further treatment of the skin disease could be managed by the medical staff inhouse. Only few consultations could not be solved virtually and were referred to local physicians for face-to-face consultations or procedures.

**Discussion:**

Teledermatological care for prisoners effectively supports the inhouse medical resources of prisons. Our interdisciplinary approach enables general practitioners and medical staff of the respective prison to manage the case and shortens the time period until therapy starts.

## Introduction

European prisons hold over half a million inmates (807,394 in 2019, data from the Eurostat database of the European Commission).^1^ German prisons hold up to 50,000 prisoners in open or closed custody (statistics as of October 7, 2021).^2^ Of these, more than 94.0% are men, 15.0% are older than 50 and more than one-third are of foreign background. Prisoners with a sentence of more than two years count for more than one-third of the inmates.^2^ These demographics show that medical care during the detention is important and the medical care in detention should reach equivalent status to the general population as stated in national and international laws and principles (German penal execution act [Strafvollzugsgesetz];^3^ EU Charter of Fundamental Rights of the European Union, Article 35: Health care).^4^

For medical care, internal medical resources are assigned to each prison. However, external physicians, mostly general practitioners (GPs) support the inhouse facilities. Additionally, every federal state of Germany runs at least one hospital for prisoners, but there are no dermatology facilities in any of the clinics run by the judicial authorities.

For specialist medical issues, either external doctors have to be requested by the prison or the prisoner has to be transported requiring time-consuming procedures, personnel and organizational effort, and financial resources.^5^ The aim to enable equivalent healthcare for prisoners compared with the people in the country is implemented as ethical standard in the recommendations of the Council of Europe, in concordance with the recommendations of the World Health Organization and the United Nations Office on Drugs and Crime.^6,7^

According to the health policy of the Council of Europe, in most European countries the federal ministry of justice is responsible for the healthcare of prisoners and should establish conditions that safeguard the health of prisoners.^8–10^ Therefore, time-saving and efficient models, for example, for access to specialized medical support, are needed. Telemedicine can support the access to healthcare and specialized medicine. Previous studies have shown first indications of the value telemedicine can provide to improved healthcare for prisoners.^11^ However, data from Germany were yet lacking.

In 2019, the German amendment to the regulations for remote treatment opened telemedical treatment of patients without personal contact and enabled a project of the ministry of justice in Baden-Württemberg, which tested telemedical care in prisons for the medical specialties general medicine and psychiatry.^12,13^ This model was extended to other prisons in additional federal states of Germany.

The aim of this study was to (1) characterize teledermatological care provided to the German prisons and (2) get evidence on the value of such extended care with focus on the need for dermatological expertise in this setting.

## Materials and Methods

### TELEDERMATOLOGICAL INTERVENTION

**Fig. 1.**
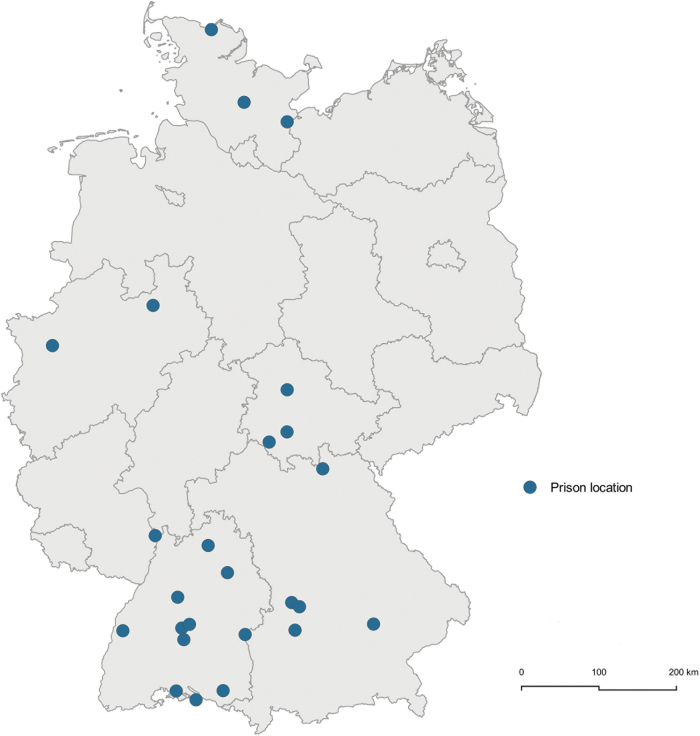
Map of Germany showing the prisons requesting dermatological teleconsultation from February 2020 until July 2021 (Geodata: © BKG, © QGIS 3.16).

Since the beginning of 2020, the dermatology team of the Institute for Health Services Research in Dermatology and Nursing (IVDP) in cooperation with the department of general practice and primary care, both at the University Medical Center Hamburg-Eppendorf (UKE), Germany, offered weekly consultation hours to prisons in Germany and joined the pilot project of Baden-Württemberg for telemedicine in prisons. These teledermatological consultations were extended from prisons in Baden-Württemberg to other federal states. Starting from five prisons in Baden-Württemberg, the service is used up to now from 25 prisons in five (out of 16) federal states in Germany with steadily rising numbers (*Fig. 1*).

The medical prison staff with the primary physician in charge of the respective institution started a request for dermatological consultation through a standardized form that the prison uploaded up to 2 h before the start of the virtual clinics to the cloud of the service provider A+ Videoclinic. On this digital request form, the prison staff had the opportunity to indicate whether it was a first onset, recurrent or chronic disease, and the duration of the skin disease. The entries could be made in an open form. Each request was managed by a qualified primary care physician and based on anonymized information regarding onset, intensity, and character of the skin problem.

There was also the possibility to upload an unlimited number of photographs of the case as well as dermatoscopic pictures before the consultation in store and forward mode. The request form included the voluntary option to state a preliminary diagnosis to indicate the need for counseling.

For the live visit on both sides, Cisco Webex devices with 1080p resolution and additional LED Heine cameras for 10–16-fold magnification and polarized photographs were installed. The prisoner had the option to join the teleconsultation live or to be represented by the medical prison staff with the store and forward information of the request form and further information during the video consultations. There was the option to involve a translator on request.

An important addition to our dermatology consultation was the interdisciplinary support by a GP attending the round simultaneously with our team, in charge for medical internal conditions, for special medications, for example, chronic infections such as hepatitis, or HIV therapies. Usually in the case of laboratory follow-up or further medical conditions other than skin problems, the GP followed the case and in some federal German counties he was able to access the electronical medical record of the prisoner, whereas our dermatology team could keep the anonymity of the patient.

Since the beginning, the weekly virtual clinics were regularly performed and the attending dermatologist, the attending GP, the patient, and the medical staff of the prison joined the round in variable settings regarding the personnel of the prison. For acute questions, the staff at the prisons could receive telemedical support at short notice, including individual enquiries within one working day. Regularly, the staff of the prison attended the consultation with at least one person, and in the majority of cases the prisoners used the possibility for their live self-presentation.

Data generated within the consultation were added to the request form and uploaded directly after the consultation enabling access to the documentation for the prison. The request forms and the photographs are saved by the number provided by the prison.

### DATA

For this study, we retrieved all data from the documented request forms from February 2020 until July 2021. Data extraction was conducted one year after implementation to avoid disruptions of results by technical or organizational issues during the implementation phase.

Data included:

- clinical symptoms and anamnestic information of the skin disease,
- patients' demographic data (age and gender),
- preliminary diagnosis and questions of the inhouse medical team of the prison regarding the case (e.g., special issues such as occupational or infectious matters, quarantine, or isolation),
- documentation of the consultation including details to onset and clinical appearance of the skin disease, and
- further anamnestic information retrieved from the virtual presentation of the case and dermatological diagnosis and suggestions for treatment.

The ethics committee of the Medical Association of Hamburg confirmed that there was no further ethic approval necessary for retrospective analysis of anonymized data in accordance with the ethical standards of the responsible committees (institutional or regional) and with the Helsinki Declaration of 1975, as revised in 1983.

### ANALYSIS

We conducted descriptive analysis of all variables with SPSS v. 26 (IBM, Armonk, NY) including demographic (e.g., age and gender) and medical data (e.g., diagnosis, suggestions for therapy, and further management of the skin disease). Thereby, we categorized open entries, for example, therapy modalities in categories such as topical or systemic therapy.

We conducted subgroup analysis for infectious diseases regarding duration of the disease till request and causative germ.

## Results

### DESCRIPTION OF THE REQUESTS

The majority of cases (98.0%) were performed during the regular weekly teleclinics and only a few (2.0%) on urgent demand within 24 h. All cases were initiated by the primary care physician of the individual prisoner. They often used the option for uploading photographs in store and forward mode before the teleclinic consultation as 90.5% of the case forms were accompanied by photographs. In 55 cases, they sent more than five photographs. In four of all cases, the photographs were not in focus so that the staff of the prison had to reload additional photographs.

Cases with first onset of the skin problem dominated the requests with 56.5%. It took 1.8 months on average from first onset until the teledermatological request. For all cases including chronic and recurrent skin problems, the average onset of the skin disease and request for dermatological expertise were three months. A total of 34 requests did not provide a definite date for onset of the skin problems (*Tables 1 and 2*).

**Table 1.** Characteristics of the Requests for Dermatology Teleconsultation

CHARACTERISTICS OF REQUESTS FOR VIDEO CONSULTATION	PATIENTS, ***n*** (%)
Gender (*n* = 200)	
Male	192 (96.0)
Female	8 (4.0)
Age (*n* = 175)	
Mean = 33.3 ± 13.0	
Median = 31 (range 16–80)	
Consultation hour (*n* = 200)	
Regular weekly teleclinic	196 (98.0)
Acute request within 24 h	4 (2.0)
Course of disease (*n* = 199)	
First onset of skin problem (median existence of skin disease before request 1.8 months; mean till request	113 (56.8)
Recurrent skin problem	80 (40.2)
Chronic skin disease	6 (3.0)
Type of request (*n* = 200)	
Request for diagnosis	138 (69)
Request for therapy recommendations	174 (87)
Concordance between prediagnosis on request form of the prison and diagnosis in video consultation by dermatologist (*n* = 177)	101 (57.1)
Photographs sent per request before video consultation (*n* = 181)	
Mean ± SD = 4.3 ± 2.9	
Median = 4 (range 1–15)	

SD, standard deviation.

**Table 2.** Time Since Onset of Skin Disease Before Request for Video Consultation (in Months)

	PATIENTS (***n***)	TIME (MEAN ± SD)	TIME (MEDIAN)	TIME (RANGE)
Consultation in general	162	10.3 ± 26.9	3	0.1–240
in cases with first onset	100	3.8 ± 6.5	1.8	0.1–36
in viral infectious infections	9	3.1 ± 4.0	1.2	0.2–12
in bacterial infections	8	3.6 ± 3.9	2.5	0.2–11
in mycotic infections	15	6.8 ± 10.7	2.0	0.2–36

The prison staff indicated “unsure” about the diagnosis on the form in 43.5% of the cases. Mainly diagnosis and/or therapy recommendations were requested. The concordance between preliminary diagnoses by prison staff and diagnoses by the teledermatologist was at 57.1%.

**Fig. 2.**
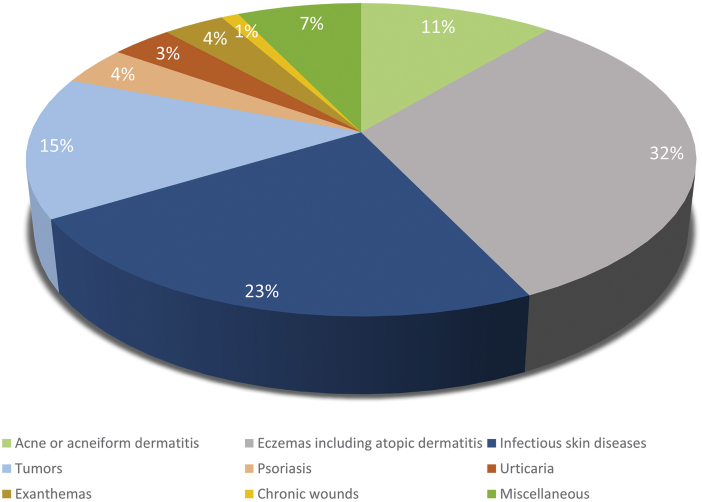
Spectrum of diagnoses.

The spectrum of skin diseases was dominated by inflammatory skin diseases and eczemas were present in almost one-third of the cases (31.8%); acne or acneiform dermatitis formed 11.1% of the requests (*Fig. 2*). Therapy for infectious skin diseases such as condyloma, fungi, or bacterial superinfections was frequently asked and covered almost a quarter of all cases (23.3%). Regarding viral skin infections (e.g., condyloma and verrucae), the consultation was performed in average 1.2 months after indicated onset of the disease with a wide range of months for fungal infections (*Table 2*).

Tumors were mostly requests for exclusion of malignancies in small skin lesions such as fibromas, angiomas, or nevi. Miscellaneous cases included, for example, dermatitis herpetiformis, Darier's disease, vitiligo, alopecia, lupus erythematosus, or cutis gyrata, but were single cases.

The symptom scores for itch and pain were documented in 194 cases. The average for itch was 3.4 on a rating scale from 0 to 10. More than one-third of the patients indicated moderate-to-severe itch (visual analog scale [VAS] ≥5) at the time of request. In addition, 7.7% had moderate-to-severe pain (VAS ≥5). Looking into the cases individually, 11 of the 15 patients with moderate-to-severe pain already received topical or systemic treatment before consultation, four patients did not start treatment beforehand. Thirteen of the patients indicating 5 and above on the itch-VAS did not report topical or systemic treatment of their skin problem before consultation. The request forms did not include further scores such as the Dermatology Life Quality Index.

In the teledermatological consultation, we recommended topical therapy in 84.0% of cases (168 of 200; *Table 3*). More than a quarter of those had no previous topical treatment for the skin disease requested, and 73.8% (124 of the 168 cases) already received topical pretreatment.

**Table 3.** Burden of the Skin Disease for the Patient

SUBJECTIVE SYMPTOMS OF CASE AT REQUEST (***n*** = 194)	PATIENTS, ***n*** (%)	MEAN ± SD	MEDIAN
VAS itch		3.4 ± 3.2	3
≥5	77 (39.7)		
<5	117 (60.3)		
VAS pain		1.0 ± 1.9	0
≥5	15 (7.7)		
<5	179 (92.3)		

VAS, visual analog scale (range from 0 to 10).

For 32.5% of all requests, we recommended systemic therapy. All of these cases already received topical treatment before the teleconsultation, and more than half of these cases already had systemic therapy that we adjusted to the present skin situation. For 19.0% of all requests (38 of 200), we recommended additional preventive or behavioral management for the skin disease. A few cases (3.5%; 7 of 200) needed further diagnostic procedures that had to be performed outside the prison, for example, allergy tests, biopsies, and surgery. Only 1.5% cases (3 of 200) did not need any therapy, for example, just confirmed a benign tumor (*Table 4*).

**Table 4.** Recommendations for Therapy with Teleconsultation (*n* = 200)

	PATIENTS, ***n*** (%)
Topical therapy	168 (84.0)
without previous topical therapy	44 (26.2)
Systemic therapy	65 (32.5)
without previous systemic therapy	38 (58.5)
Preventive/behavioral measures	38 (19.0)
Need for further diagnostic procedures	24 (12.0)
No measures required	3 (1.5)

The rate for follow-up requests was low. Within the interval observed from February 2020 until July 2021, we documented only eight requests for follow-up after the first consultation; this was performed with a second request during regular teleconsultation. Overall, 184 cases could be finalized with a single telemedical consultation and the medical prison staff. The GP on demand took over without further information for us.

## Discussion

This is the first study demonstrating clinical usefulness of teledermatological consultations in German prisons. The majority of cases were performed during a regular weekly teleconsultation and only a few cases (3 of 200) on urgent demand within 24 h. The skin diseases presented reflected the average spectrum of skin diseases seen in a regular outpatient setting with emphasis on inflammatory skin diseases of younger patients.^14^ The spectrum of requests included assessments for risks of infectious diseases. The timely answer to these requests is of key interest in these facilities with close contact of inhouse staff and prisoners in arrest and transport.

A significant number of cases had a first onset of the skin problem or acute recurrence of previously known skin diseases. The majority needed a diagnosis for further management, and the prisons used the option to seek help in case of uncertainty. The preliminary diagnosis stated by the medical staff of the prison was confirmed in only about half of all requests by the dermatological video consultation. This finding underlines the need for specialized expertise and the importance for timely access to interdisciplinary expertise to support the in-house facilities of a prison.

Summarizing the symptom score of the patients, there is a significant burden of skin problems reflected by the scores for itch or pain. Some patients with moderate-to-severe pain did not start treatment of the disease before consultation and a significant number of the patients indicating severe itching did not report topical or systemic treatment of their skin problem before consultation. These symptom scores are based on self-reporting by the prisoners and do not include information about the timespan for the prisoners spent in prison before request of their symptoms.

Therefore, the value for interpretation of these scores is limited. But it shows a need for dermatological consultations also inside the prison in general. Telemedicine facilitates the access to medical expertise and the data support the need for timely approach to dermatological expertise like this teleconsultation. The average duration for all cases since onset of the skin disease and request for dermatological expertise was three months, that for skin problems with first onset of the disease was 1.8 months.

This again does not necessarily reflect an onset inside the prison but can also mean the presence of the disease already before arresting the patient. The need for dermatological consultation in the prison with timely presentation of the skin problem is obvious and the time from onset until presentation is slightly longer than visits from outpatients in ambulatory settings in Germany.^15,16^ In addition, the option to address any medical need might help prisoners to get medical services that they might not have reached in normal life. The medical services for prisoners are covered by the federal states and this means medical therapies outside the conditions of health insurances for the average population.

The majority of cases asked for therapy suggestions, and for about one-third of all requests we recommended systemic therapy. More than half of those cases had already systemic treatments before consultation and consequently moderate or severe skin disease. This reflects the severity of cases and the need for treatment. Only few cases had no need for any treatment or further procedures.

The predominant number of cases was closed after one consultation and only a few cases asked for follow-up, which could be performed with another teleconsultation. The support by a GP involved simultaneously is extremely helpful for taking over regular laboratory controls or follow-up of medication. This shows a very effective way to offer specialized expertise to this particular setting of arrested patients in a time and resource saving way. The alternative with a transport to a facility outside the prisons means additional security issues and needs thorough management and planning.

The presentation in an outdoor institution is also an exposition of the prisoner that can be avoided by our pseudonymized request form and the possibility to upload dermoscopic photographs and close-up photographs instead of overviews with identification of the person. Although digital sources for information are state of the art, there are conditions that cannot be solved with a virtual visit and had been recommended to further investigations at local facilities, such as biopsies, dignity of tumors, and palpable lesions. The procedure follows national guidelines published in 2020.^17^

In this video setting, patients have the choice to present themselves live or to be represented by the medical staff on site that respects individual emotional needs for anonymity and can regard organizational circumstances of the penitentiary. The medical colleagues of the prisons use the case presentation for background information about the skin disease, and we enable the colleagues on site to understand and overtake the case that saves time and personal in follow-up. This is especially important for severe skin diseases with need for systemic therapies and an anticipated potential for recurrences and the need for control of therapy, for example, lupus therapy.

Our consultation includes a discussion about options for therapy and the prison and the prisoner can add information that is helpful for the anamnestic background of the case. The GP involved is able to look into the electronical file of the patient; can help with nondermatological treatments as there are regularly antipsychotic medication, hepatitis treatment, and drug addict programs; and can start the therapy recommendations at the moment of the live consultation; this is very time saving and the prisoner receives therapy without delay. This is a new approach to specialized medical expertise with a close interdisciplinary exchange that differs from other models providing dermatological teleconsultation for prisoners in other countries.^18^

The request form and the photographs are filed and, therefore, enable us to recall cases and to document the cases, the patient is pseudonymized and we do not see the entire file of the prisoner that leaves us on a neutral professional approach to the skin problem.

This model for an interdisciplinary teleconsultation offers dermatological expertise to prisons in Germany in an effective time- and resource-saving way to ensure specialized care in facilities with difficult access to medical expertise. We have shown a pragmatic way to perform dermatological teleconsultations. The high symptom scores, the spectrum of skin diseases, including moderate-to-severe cases, and the therapies recommended support the importance of regular reliable weekly teleconsultations for the prisoners to enable early intervention in progressing skin diseases.

In conclusion, our model offers numerous possibilities for various medical disciplines to combine telemedical strategies with specialized expertise and shows an efficient way to support interdisciplinary teamwork.
